# miR-720 is a downstream target of an ADAM8-induced ERK signaling cascade that promotes the migratory and invasive phenotype of triple-negative breast cancer cells

**DOI:** 10.1186/s13058-016-0699-z

**Published:** 2016-04-02

**Authors:** Sonia G. Das, Mathilde Romagnoli, Nora D. Mineva, Sophie Barillé-Nion, Pascal Jézéquel, Mario Campone, Gail E. Sonenshein

**Affiliations:** Department of Developmental, Molecular and Chemical Biology, Tufts University School of Medicine, Boston, MA 02111 USA; INSERM U892, IRT-UN, 8 quai Moncousu, 44007 Nantes Cedex, France; Institut de Cancérologie de Nantes, Centre de Lutte Contre le Cancer René Gauducheau, Boulevard Jacques Monod, 44 805 Saint-Herblain-Nantes Cedex, France; Present address: Institut Curie, Centre de Recherche, UMR 144, 26 Rue d’Ulm, 75248 Paris, France

**Keywords:** miRNA, Triple-negative breast cancer, ADAM8, miR-720, ERK

## Abstract

**Background:**

ADAM8 (a disintegrin and metalloproteinase 8) protein promotes the invasive and metastatic phenotype of triple-negative breast cancer (TNBC) cells. High ADAM8 expression in breast cancer patients is an independent predictor of poor prognosis. Here, we investigated whether ADAM8 regulates specific miRNAs, their roles in aggressive phenotype, and potential use as biomarkers of disease.

**Methods:**

Microarray analysis was performed on RNA from MDA-MB-231 cells after transient ADAM8 knockdown using TaqMan miRNA cards. Changes in miRNA levels were confirmed using two *ADAM8* siRNAs in TNBC cell lines. Kinase inhibitors, β1-integrin antagonist antibody, and different forms of ADAM8 were employed to elucidate the signaling pathway required for miR-720 expression. miR-720 levels were modulated using a specific antagomiR or a mimic, and effects on aggressive phenotype of TNBC cells were determined using Boyden chamber and 3D-Matrigel outgrowth assays. Plasma was isolated from mice before and after implantation of MDA-MB-231 cells and analyzed for miR-720 levels. Serum samples of TNBC patients were evaluated for their ADAM8 and miR-720 levels.

**Results:**

We identified 68 miRNAs differentially regulated upon ADAM8 knockdown, including decreased levels of secreted miR-720. Ectopic overexpression of wild-type ADAM8 or forms that lack metalloproteinase activity similarly induced miR-720 levels. The disintegrin and cysteine-rich domains of ADAM8 were shown to induce miR-720 via activation of a β1-integrin to ERK signaling cascade. Knockdown of miR-720 led to a significant decrease in migratory and invasive abilities of TNBC cells. Conversely, miR-720 overexpression rescued these properties. A profound increase in plasma levels of miR-720 was detected 7 days after TNBC cell inoculation into mouse mammary fat pads when tumors were barely palpable. Concordantly, miR-720 levels were found to be significantly higher in serum samples of TNBC patients with high ADAM8 expression.

**Conclusions:**

We have shown for the first time that miR-720 is induced by ADAM8 signaling via ERK and plays an essential role in promoting the aggressive phenotype of TNBCs. miR-720 is elevated in serum of patients with ADAM8-high TNBC and, in a group with other miRNAs downstream of ADAM8, holds promise as a biomarker for early detection of or treatment response of ADAM8-positive TNBCs.

**Electronic supplementary material:**

The online version of this article (doi:10.1186/s13058-016-0699-z) contains supplementary material, which is available to authorized users.

## Background

Triple-negative breast cancers (TNBCs), which lack expression of the estrogen receptor alpha (ERα), progesterone receptor, and human epidermal growth factor receptor 2 (HER2), are particularly aggressive and clinically challenging to manage due to the absence of these common therapeutic targets [1]. TNBC is responsible for approximately 25 % of all breast cancer deaths, although it represents only 15 % of patients diagnosed with invasive breast cancer. TNBC patients have a poorer clinical outcome, and experience a higher rate of distant recurrence (34.0 % versus 20.4 % in other breast cancers) [2]. In consequence, they have a lower 5-year overall survival rate than patients with other breast cancer subtypes [3]. Younger women and women of African-American descent are at a particularly high risk of TNBC [4]. Therefore, there is a critical need to understand the molecular mechanisms promoting the aggressive phenotype of TNBCs and to identify additional prognostic markers to improve early clinical diagnosis of primary or recurrent disease [5].

MicroRNAs (miRNAs) are a class of endogenously expressed, evolutionarily conserved, small noncoding RNAs, which play crucial regulatory roles in a variety of normal cellular processes [6]. Their aberrant expression in cancer has been implicated in various signaling pathways affecting tumor initiation, growth, invasion, and metastasis [7]. For example, miR-10b has been shown to promote metastasis of 4T1 cell line-derived breast tumors in a mouse mammary model [8]. In contrast, miR-let-7 suppresses breast cancer cell migration and invasion, in part through downregulation of C-C chemokine receptor type 7 [9], while in lung cancer, low miR-let-7 levels lead to higher expression of RAS protein [10]. Blenkiron et al. showed that miR-155 is differentially expressed in ERα– versus ERα + tumors, and is overexpressed in breast tumors compared to normal breast tissue [11]. Differential expression of six miRNAs (miR-142-3p, miR-505*, miR-1248, miR-181a-2*, miR-25* and miR-340*) was found to accurately discriminate between tumors from BRCA1/2 mutation carriers and noncarriers [12]. Furthermore, miRNAs are stable in both primary tumors and in the circulation, leading researchers to propose their use as biomarkers for cancer diagnosis and prognosis. The importance of circulating miRNAs as biomarkers for breast cancer has been documented in several studies [13–16]. For example, Zhu and coworkers showed that miR-155 may be differentially expressed in the serum of women with hormone-sensitive versus hormone-insensitive breast cancer [15]. Madhavan et al. demonstrated that breast cancer patients that are positive for circulating tumor cells (CTCs) versus patients negative for CTCs had significantly higher levels of miR-141, miR-200a, miR-200b, miR-200c, miR-203, miR-210, miR-375, and miR-801 in their plasma [13]. While considerable effort has been invested in understanding how miRNAs regulate various genes, much less is known about the regulation of miRNA expression.

ADAM8 (a disintegrin and metalloproteinase 8) is a transmembrane protein that belongs to the ADAM family of proteins that mediate cell adhesion, cell migration, and proteolysis of a variety of substrates in the extracellular matrix [17]. ADAM8 is synthesized with a signal sequence along with five domains, namely the prodomain (PRO), and the metalloproteinase (MP), disintegrin (DI), cysteine-rich (CRD), and epidermal growth factor (EGF)-like (ELD) domains. It also has a transmembrane region and a cytoplasmic tail. The MP domain is catalytically active [18, 19] and can shed various cytokines and their receptors. The DI and CRD domains of ADAM proteins have been suggested to bind integrins and other receptors, and mediate cell adhesion. This adhesion can sometimes occur via an RGD sequence [20]; however, ADAM8 lacks this sequence and Stone et al. [21] have proposed that the three-dimensional (3D) structure of the disintegrin loop, and not its sequence, may play an important role in mediating these interactions. ADAM8 is synthesized as a 120-kDa proform, which can dimerize or multimerize, and autocatalytically clip off its prodomain, leaving an active membrane-associated metalloprotease of 90-kDa [22]. Active ADAM8 can be further processed by the release of the MP domain into the extracellular matrix, leaving behind a 60-kDa membrane-associated remnant form. The cytoplasmic tail of ADAM8 is relatively long and has a conserved potential SH3 ligand domain, similar to ADAM9 [23]. The natural ligands of the ADAM8 cytoplasmic domain have not yet been identified, but it is likely that the tail has signaling potential.

Recently, our laboratory has shown that ADAM8 is highly expressed in breast tumors, especially in TNBC, compared to normal tissue and its level correlates with poor patient outcome [24]. Furthermore, ADAM8 was detected by immunohistochemistry in 48 % of all breast cancer-derived metastases. Knockdown of ADAM8 in TNBC cells decreased their ability to migrate, to invade through Matrigel in a Boyden chamber assay, and to form branched colonies in 3D-Matrigel outgrowth assays in vitro. In an orthotopic mouse model, tumors derived from human TNBC cells with ADAM8 knockdown failed to grow beyond a palpable size due to impaired angiogenesis, and showed greatly reduced ability to metastasize [24]. Mechanistic studies identified two major ADAM8 functions: (1) promoting angiogenesis through release of VEGF-A and other pro-angiogenic factors; and (2) activating β1-integrin on the cancer cells needed for intravasation and extravasation allowing for tumor dissemination and metastasis. Significantly, treatment with an anti-ADAM8 antibody targeting its extracellular MP and DI domains reduced primary tumor burden and metastases in mice [24]. Given the key role ADAM8 plays in promoting invasion and metastasis of TNBCs, here we tested the hypothesis that ADAM8 mediates the aggressive phenotype of TNBC cells through regulation of specific miRNAs. Using ADAM8 knockdown strategies, 68 miRNAs were identified in MDA-MB-231 TNBC cells, including miR-720 which is overexpressed in several cancers [25] and secreted from TNBC cells. miR-720 was shown to be induced by ADAM8 via a β1-integrin to ERK signaling cascade, and to mediate signals that promote the invasive and migratory phenotype of TNBC cells in culture. In an orthotopic mouse model, miR-720 was detectable in the serum of mice bearing ADAM8-expressing tumors. Importantly, miR-720 levels were elevated in serum samples of TNBC patients with high ADAM8 expression. Overall, these studies suggest that miR-720 plays an essential role in the aggressive phenotype of ADAM8-positive TNBCs and may serve in a group of miRNAs as a biomarker for early detection of recurrence and treatment efficacy in ADAM8-positive TNBC patients.

## Methods

### Antibodies and inhibitors

The ADAM8 antibody (B4068) used for Western blotting was purchased from LifeSpan Biosciences. Antibodies against β-actin (AC-15) and β-tubulin (TUB 2.1), and the ERK inhibitor FR180204 (SML0320) were obtained from Sigma-Aldrich. The antibody to detect specific ERK 1/2 phosphorylated forms (pERK1/2) (9101) was obtained from Cell Signaling Technology. The β1-integrin antibody (552828) and its control isotype matched IgG2A (SC3883) were purchased from BD Biosciences and Santa Cruz Biotechnology, respectively. The anti-ADAM8 antibody MAB10311 and its control isotype-matched IgG1 (MAB002) were from R&D Systems.

### Cells and culture conditions

The MDA-MB-231 and Hs578T human TNBC cell lines and the human umbilical vein endothelial cell (HUVEC) line were purchased from the American Type Culture Collection (ATCC) and maintained in media recommended by ATCC. Stable clones of ADAM8 short hairpin RNA (shRNA) (shA8-20) and control shRNA (shCtrl-3) MDA-MB-231 cells expressing green fluorescent protein were isolated as described previously [24], and kindly provided by Joerg Bartsch (Philipps University, Marburg, Germany). The identity of the MDA-MB-231 cell line and the shA8-20 and shCtrl-3 MDA-MB-231 clones was authenticated using short tandem repeat analysis (Genetica DNA Laboratories), which showed 100 % identity with the MDA-MB-231 cell line of ATCC. The inflammatory breast cancer cell line SUM-149 was kindly provided by Stephen Ethier (University of Michigan Medical School, Ann Arbor, MI, USA) and maintained as previously described [26]. HEK-293 cells were kindly provided by Nader Rahimi (Boston University School of Medicine, Boston, MA, USA). All cell cultures were confirmed to be free of mycoplasma contamination using polymerase chain reaction (PCR) (VenorGeM Mycoplasma Detection Kit, Sigma). To test for the effects of inhibiting ADAM8 activity, cells were treated either with siADAM8 (see below) or with 20 μg/ml anti-ADAM8 antibody MAB10311, or control isotype IgG1 MAB002. To test for the effects of inhibition of β1-integrin signaling, cells were treated with 10 or 20 μg/ml of either antagonist β1-integrin antibody or control isotype (IgG2A rat). To inhibit ERK signaling, cells were treated with 25 μM FR180204.

### Cloned DNA and plasmid transfection

The human full-length ADAM8 (hADAM8) cDNA (MGC:134985; Genbank:BC115404.1) and the remnant form ADAM8 cDNA were kindly provided by Joerg Bartsch as described previously [27]. The enzymatically inactive mutant of hADAM8 was prepared as described previously [28]. For transient transfection of shA8-20 cells, cultures were incubated for 48 h in the presence of 4 or 8 μg total DNA in six-well or P60 plates, respectively, with Lipofectamine 2000 (Invitrogen) transfection reagent. For transfection of HEK-293 cells, 1 or 2 μg of total DNA in Lipofectoamine 2000 was used, as indicated. Empty pcDNA3 vector (EV) or pcDNA3.1 myc-his vector (EV-3.1) DNA were used as negative controls.

### siRNA knockdown analyses

Transient RNAi-mediated ADAM8 knockdown was performed with the following short interfering RNAs (siRNAs) (Qiagen):

- *siADAM8-1* (Hs_ADAM8_6): 5′-CGGCACCTGCATGACAACGTA-3′;
- *siADAM8-2* (Hs_ADAM8_7): 5′-CTGCGCGAAGCTGCTGACTGA-3′;

AllStar negative control siRNA (Qiagen) was used as a non-silencing control siRNA (*siCtrl*). siRNAs (10 nM) were introduced in MDA-MB-231 or SUM-149 cells using Lipofectamine RNAi Max Transfection Reagent (Invitrogen) by reverse transfection according to the manufacturer’s protocol. Transfected cells were used 48 or 72 h later in functional assays.

### miRNA microarray analysis and validation studies

MDA-MB-231 cells were transiently transfected with *siADAM8-2* or *siCtrl*. Total RNA was isolated 72 h after transfection using TRIzol reagent (Invitrogen), according to the manufacturer’s protocol and quantified using a Take3 plate reader (BioTek Synergy HT). We chose 72 h post-transfection for functional assays to decrease effects introduced as a result of the transfection protocol. RNA integrity was assessed using the Agilent 2100 Bioanalyzer (Agilent) and RNA 6000 Nano LabChip kit (5065–4476). RNA with an RNA integrity number (RIN) >9.0 was considered good quality. Array analysis was performed using the TaqMan Array Human MicroRNA A + B cards v3.0 (Life Technologies, 4444913) according to the manufacturer’s protocol. Briefly, 1 μg total RNA was amplified and cDNA prepared using Megaplex Reverse Transcriptase (RT) Human Pool Set v3.0 primers (Life Technologies, 4444745) and TaqMan microRNA reverse transcription kit (Invitrogen, 4366596). Subsequently, 6 μl of the cDNA preparation was diluted with TaqMan Universal PCR Master Mix (450 μl) and nuclease free water (444 μl) and loaded on the 384-well TaqMan low-density array card. The cards were then centrifuged to distribute the cDNA samples in the reaction wells using a refrigerated Sorvall and Heraeus bucket centrifuge at 1200 rpm (331 × g) for two 1-min runs. The plate was then sealed and the real-time PCR was carried out on an Applied Biosystems 7900HT Real-Time PCR system. The data were analyzed using SDS software. Relative miRNA expression was calculated by comparing MDA-MB-231 cells with ADAM8 knockdown to cells with the control siRNA. This experiment was performed in duplicate and miRNAs that showed greater than twofold change in both replicates were selected for study.

### Mammary fat pad mouse model

All animal work was performed in accordance with a protocol approved by the Institutional Animal Care and Use Committee of Tufts University and Tufts Medical Center. Blood was collected from 6-week-old female nonobese diabetic/severe combined immunodeficient (NOD/SCID) mice (Jackson Laboratory) using submandibular bleeding and plasma isolated and processed as described below. The next day, 2.5 × 10^6^ shCtrl-3 MDA-MB-231 cells in 40 μl 50 % Matrigel (BD Biosciences, CB-40230A) solution (1:1 dilution of Matrigel with DMEM medium) were implanted in the fourth inguinal mammary fat pad. Primary tumor growth was monitored by caliper measurement twice a week. Tumor volumes were calculated as (length × width^2^)/2. At the indicated times, blood was collected and processed as above. Mice were sacrificed after 3–4 weeks when tumors derived from shCtrl-3 cells had reached a volume of ~1 cm^3^. Tumors were dissected, photographed, weighed and flash frozen.

### Quantitative RT PCR analysis of cell RNA

RNA was isolated from cells using TRIzol reagent and DNA prepared using TaqMan MicroRNA Reverse Transcription Kit (Invitrogen, 4366596) according to the manufacturer’s protocol. Expression of miRNAs was assessed by quantitative reverse transcription PCR (RT-qPCR), using *U6* snRNA (Invitrogen, 4427975) as the control. Single tube TaqMan assays (Invitrogen, 4427975) were obtained for all miRNAs of interest (hsa-miR-30d*, hsa-miR-181a-2*, hsa-miR-29c, rno-miR-29C*, hsa-miR-93*, hsa-miR-520c-3p, hsa-miR-130b*, hsa-miR-720, hsa-miR-106*b, hsa-miR-98, and hsa-miR-20a*) and qPCR was carried out as follows: 95 °C for 10 min, then 40 cycles of 95 °C for 15 s and 60 °C for 60 s. All analyses were performed in triplicate and the data were normalized to *U6* snRNA. Average fold-change ± SD in miRNA levels relative to those in control untreated cells (set to 1) are presented.

### RNA extraction from mouse plasma

Following isolation of mouse blood via submandibular bleeding, 25 μl of a 10-mM EDTA solution was added to individual samples to prevent coagulation. The samples were stored on ice and centrifuged at 1300 g for 20 min at 4 °C. Supernatants were collected and lysed as recommended in the manufacturer’s protocol for the miRneasy serum/plasma kit (Qiagen, 217184). Subsequently, 3.5 μl of *C. elegans* miR-39 (Qiagen, 219610) (160 nM) was added as a spike in each sample to control for miRNA recovery. RNA was then isolated as per protocol. Reverse transcription was carried out using the miScript II RT kit (Qiagen, 218161). miRNA expression was assessed by qPCR, and values normalized to the control *C. elegans* miR-39 (Ce_miR-39_1, MS00019789, Qiagen). The miScript primer assay was used for qPCR of miR-720 (Hs_miR720_1, MS00014833, Qiagen) as follows: 95 °C for 15 min, then 45 cycles of 94 °C for 15 s, 55 °C for 30 s and 70 °C for 30 s. Average fold-change ± SD in normalized miR-720 miRNA levels relative to those in control untreated mice are presented.

### Exosome isolation and RNA extraction

Exosome isolation was performed essentially as published previously [29]. Briefly, cell lines were cultured in ten P100 plates each until they reached 50–70 % confluency. The culture media for MDA-MB-231, shA8-20 and shCtrl-3 cells were then replaced with serum-free media, whereas, for SUM-149 cells which require serum for their viability, exosome-depleted FBS media was used. After 72 h, supernatants were collected, centrifuged at 2000 × g for 20 min to remove debris and filtered using a 0.22-μm filter. Exosomes were isolated by ultracentrifugation at 100,000 × g for 70 min. The exosome pellet was washed with PBS and RNA isolated using the miRCURY RNA isolation kit-cell and plant (Exiqon, 300110). Isolated RNA was subjected to Agilent 2100 Bioanalyzer (Agilent) analysis using a RNA 6000 Nano LabChip kit (5065–4476), which confirmed the lack of rRNA in the miRNA samples. Levels of miR-720 expression, determined by RT-qPCR, are presented ± SEM from two independent experiments.

### miRNA knockdown and overexpression

MDA-MB-231 and SUM-149 cells were transfected in six-well plates with 50 nM miR-720 mirVana miRNA inhibitor (Invitrogen, 4464084 (hsa_miR_720, assay id: MH13574)) or negative-control (Invitrogen, 4464076) oligonucleotides using Lipofectamine RNAi Max (Invitrogen). After 16 h, fresh media was added and the cells were collected 48 h after transfection for functional analysis. To overexpress miR-720, ADAM8 knockdown MDA-MB-231 cells (shA8-20 clone) were plated a day prior to transfection. When cells reached 80 % confluency, cultures were transfected overnight with 200 pmol miR-720-mimic (Invitrogen, 4464066 (assay id: MC13574)) or the negative control mimic (Invitrogen, 4464058) using Lipofectamine 2000. The transfection media were replaced with fresh media and cells harvested 48 h after transfection for functional analysis.

### Western blot analysis

Whole-cell extracts (WCEs) were prepared using RIPA buffer (50 mM Tris pH 7.6, 150 mM NaCl, 1 % NP-40, 0.1 % SDS, 5 mM EDTA, 1 % sodium sarkosyl) supplemented with protease and phosphatase inhibitors [30], and 5 mM EDTA and 10 mM phenanthroline to inhibit the autocatalytic activity of ADAM8. Lysates were sonicated and centrifuged at 16,000 g for 15 min. Protein concentration in the supernatants was determined using DC Protein Assay Reagent (BioRad). Samples (25 μg) were subjected to immunoblotting as previously described [27]. Either β-actin or β-tubulin was used as the loading control.

### ATP assay

As a measure of cellular metabolism and therefore cell growth, ATP levels were assessed using an ATPlite luminescence ATP detection assay system (Perkin Elmer), as described previously [31]. Briefly, cells (3000 cells/100 μl for MDA-MB-231 and shA8-20, or 8000 cells/100 μl for SUM-149) were plated in 96-well plates. After 24 h, an equal volume of APTlite 1Step luminescence reagent was added and luciferase activity measured. Average ATP levels, for triplicate samples, are presented relative to the control condition set to 1 (± SD).

### Matrigel outgrowth, migration/invasion and transendothelial migration assays

Matrigel outgrowth assays were carried out as described previously [32], using 5 × 10^3^ cells plated in duplicate in 24-well plates. Cultures were incubated for 11–15 days and photographed at 10× magnification. Migration and invasion assays were performed in triplicate using polycarbonate filter Transwells (Costar) with 8-μm diameter pores, without precoating or precoated with growth factor-reduced Matrigel (BD Biosciences, 356231), respectively. For transendothelial migration, Transwells were coated with a confluent layer of HUVECs instead of Matrigel. Suspensions of 1 × 10^5^ tumor cells were layered in the upper compartment of the Transwells and incubated at 37 °C. After 24 h, cells that migrated or invaded to the lower side of the filter were quantified by crystal violet staining and OD_570nm_ determination. The negative control or control mimic condition was set to 100 % and the mean ± SD from three independent experiments is given.

### ELISA and RNA analysis of serum from TNBC patients

All clinical investigation was conducted in accordance with the principles outlined in the Declaration of Helsinki. Serum samples used in this work were provided by the Institut Régional du Cancer Nantes-Atlantique tumor bank, funded by the Institut National du Cancer (INCa) and approved by the French Minister of higher education and research (n°. AC-2008-141). Informed consent was obtained from patients to use their surgical specimens, serum, and clinicopathological data for research purposes, as required by the French Committee for the Protection of Human Subjects (CCPPRB). Ouest IV – Nantes CCPPRB approved use of serum samples for this study (6 May 2013: n°. 357/2013). This study did not need additional ethical approval.

Blood samples were collected in BD Vacutainer red-top tubes (BD Biosciences, 367837) from 37 consenting women diagnosed with TNBC in the Nantes Cancer Center (ICO, France). At the time of sampling, patients had not received any treatment in the form of surgery, chemotherapy, radiation, or endocrine therapy. Blood samples were allowed to clot for 1 h and centrifuged at 400 g for 10 min. Sera were stored at –80 °C within 2 h of being taken. Detailed patient clinicopathological characteristics are listed in Additional file 1 (Table S1). Sera were also collected from 15 consenting healthy female individuals. ADAM8 protein was measured in the serum using an enzyme-linked immunosorbent assay (ELISA) (R&D Systems) according to the manufacturer’s instructions.

Serum samples from patients (150 μl) were thawed to room temperature and processed as recommended for the NucleoSpin miRNA plasma kit (Invitrogen, 740981). Subsequently, 3.5 μl *C. elegans* miR-39 (Qiagen, 219610) (160 nM) was added as a spike in each sample to control for miRNA recovery. RNA was then isolated as per protocol. Reverse transcription was carried out using the TaqMan MicroRNA Reverse Transcription Kit (Invitrogen, 4366596). miRNA expression was assessed by qPCR, and values were calibrated to the control *C. elegans* miR-39 (Cel-miR-39-3p). Single tube TaqMan assays (Invitrogen, 4427975) for hsa-miR-720 were carried out as follows: 95 °C for 10 min, then 40 cycles of 95 °C for 15 s and 60 °C for 60 s. All analyses were performed in triplicate and the data were normalized to hsa-miR-16 (Invitrogen, 4427975). Serum levels of miR-720 in patients versus healthy individuals are presented as average fold-change ± SD.

## Results

### ADAM8 regulates miRNA expression in MDA-MB-231 TNBC cells

**Fig. 1:**
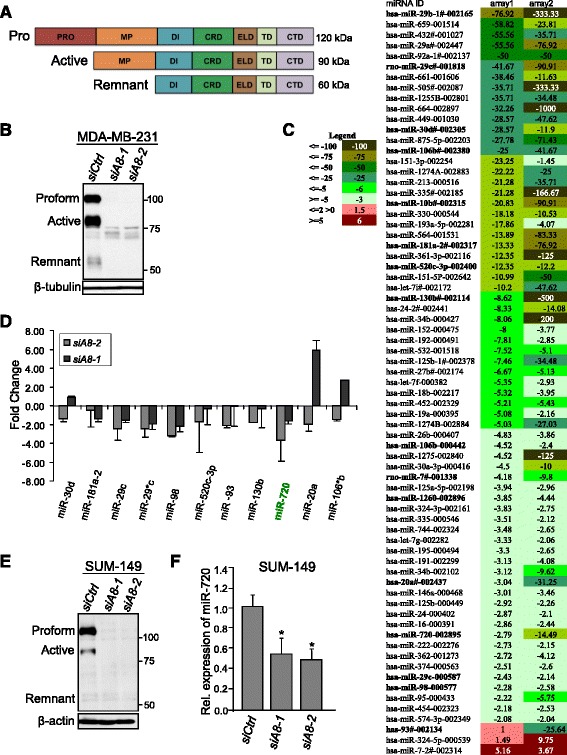
Knockdown of ADAM8 alters miRNA expression in TNBC cells. **a** Schematic representation of the various domains of the human ADAM8 protein and the different forms observed after processing in cancer cells. **b**, **c** MDA-MB-231 cells were transfected with 10 nM of either control siRNA (*siCtrl*) or the ADAM8 siRNAs *siADAM8-1* or *siADAM8-2* (*siA8-1* or *siA8-2*). After 72 h, WCEs and RNA were isolated. WCEs (25 μg) were examined by Western blotting for expression of ADAM8, and β-tubulin as a loading control. A representative blot is shown (*n* = 3). ADAM8 forms and molecular weight (MW) markers are as indicated (**b**). RNAs isolated from *siCtrl* and *siADAM8-2* cells were subjected to an RT-qPCR-based miRNA array assay and the resulting heat map representation of miRNA expression displaying a greater than twofold change presented as fold-changes in *siADAM8-*2 compared to *siCtrl* in two independent experiments (Arrays 1 and 2) (**c**). The legend for the fold-changes in the heat map is given to the left. **d** RNA from MDA-MB-231 cells transfected with 10 nM of *siCtrl, siADAM8-1* or *siADAM8-2* for 72 h were subjected to RT-qPCR for the indicated miRNAs. *siCtrl* was set to 1 and the fold changes given as mean ± SD from three independent experiments. **e** WCEs from SUM-149 cells transfected with 10 nM of *siCtrl*, *siADAM8-1* or *siADAM8-2* for 72 h were subjected to Western blotting for expression of ADAM8, and β-actin as a loading control. A representative blot is shown (*n* = 3). Positions of the proform, active and remnant forms of ADAM8 and MW markers are as indicated. **f** RNAs were isolated from SUM-149 cells transfected, as in part **e**, and samples subjected to RT-qPCR for miR-720 levels. *siCtrl* was set to 1 and relative (*Rel.*) levels of miR-720 are given (mean ± SD from three independent experiments). *P < 0.05, Student’s *t* test. *CRD* cysteine-rich domain, *CTD* cytoplasmic tail domain, *DI* disintegrin, *ELD* epidermal growth factor-like domain, *MP* metalloproteinase, *PRO* prodomain, and *TD *transmembrane domain.

ADAM8 is synthesized as a multidomain proteolytically inactive proform protein (120-kDa), which can autocatalytically clip its prodomain, leaving an active 90-kDa protein with MP activity, which can be further processed to a 60-kDa remnant transmembrane form lacking the MP domain but retaining the disintegrin domain and C-terminal region (Fig. 1a). As ADAM8 promotes the migratory and invasive phenotype of TNBC cells [24], we sought to determine whether any of these effects were mediated via downstream miRNAs. Firstly, we asked whether ADAM8 regulates miRNA expression in MDA-MB-231 TNBC cells using an RT-qPCR-based miRNA array. Seventy-two hours following transfection of MDA-MB-231 cells with either *siADAM8-1* or *siADAM8-2* or a control siRNA (*siCtrl*), WCEs were isolated and subjected to Western blotting (Fig. 1b). Both *ADAM8* siRNAs effectively reduced the levels of ADAM8 compared to the *siCtrl*. As *siADAM8-2* was previously shown to be slightly more effective in downregulating ADAM8 function in MDA-MB-231 cells [24], it was selected to knockdown ADAM8 for the miRNA array assay. RNA was isolated from two independent cultures of MDA-MB-231 cells 72 h post-transfection with *siADAM8-2*. The RNA preparations were subjected to TaqMan low-density miRNA array card analysis. The relative miRNA expression in the *siADAM8* RNA preparations compared to the *siCtrl* RNA was calculated using the SDS software. A threshold increase or decrease of twofold was used as the cut-off, and 68 miRNAs were differentially regulated greater than or equal to twofold upon ADAM8 knockdown with *siADAM8-2* (Fig. 1c). Of these 68 miRNAs, 66 miRNAs were downregulated and 2 miRNAs were upregulated. Literature analysis was performed to identify the miRNAs in this subset that had been reported aberrantly expressed in cancer [33–42], and 11 oncogenic miRNAs (oncomiRs) were identified. To confirm that these miRNAs were indeed regulated by ADAM8, RT-qPCR for these 11 miRNAs was performed using three independently isolated RNA preparations from MDA-MB-231 cells treated with *siADAM8-1* or *siADAM8-2* versus *siCtrl*. As seen in Fig. 1d, knockdown of ADAM8 with *siADAM8-2* led to reduced levels of all 11 RNAs, confirming the TaqMan low-density miRNA array card analysis. However, treatment with *siADAM8-1* led to reduced levels of only eight miRNAs (miR-181a-2, miR-29c, miR-29c*, miR-98, miR-520c-3p, miR-93, miR-130b, and miR-720), whereas three miRNAs showed increased expression, including miR-30d, miR-20a and miR-106*b (Fig. 1d), suggesting these miRNAs may not be regulated specifically by ADAM8 or may have differential regulation via splice variants. Overall, multiple miRNAs appear to be regulated by ADAM8 or a downstream mediator/pathway.

### Expression of miR-720 is induced by ADAM8

While all of the ADAM8-regulated miRNAs have been implicated in various aspects of tumorigenesis, we were particularly interested in miR-720 since it has been reported to be highly expressed and abundantly released from breast cancer cells [25, 43–45] and detected in the blood of patients with multiple myeloma [46]. Also, we found that miR-720 was highly expressed in MDA-MB-231 cells (Additional file 2: Figure S1). The transfection of MDA-MB-231 cells with *siADAM8-2* and *siADAM8-1* led to a 3.7-fold and 1.7-fold downregulation of miR-720, respectively (Fig. 1d). Recently, Lerebours et al. [25] identified miR-720 in a set of five miRNAs as a predictive marker of poor prognosis in patients with inflammatory breast cancer (IBC), which we have found also frequently express ADAM8 (data not shown). Thus, we next analyzed the effects of ADAM8 knockdown on SUM-149 cells, a triple-negative IBC line which has been characterized as a basal-like 2 (BL2) subtype line of TNBC [47] that expresses high levels of ADAM8 [24]. SUM-149 cells were transfected with *siCtrl*, *siADAM8-1* or *siADAM8-2*, and after 72 h WCEs and RNA were isolated. Western blot analysis confirmed the effective knockdown of ADAM8 by the two specific siRNAs (Fig. 1e). RT-qPCR of RNA isolated in three independent experiments indicated miR-720 levels were reduced by 2-fold and 1.7-fold, respectively, in SUM-149 cells treated with *siADAM8-2* and *siADAM8-1* compared to the *siCtrl* (Fig. 1f). Thus, knockdown of ADAM8 decreases miR-720 levels in both MDA-MB-231 and SUM-149 TNBC cells.

**Fig. 2:**
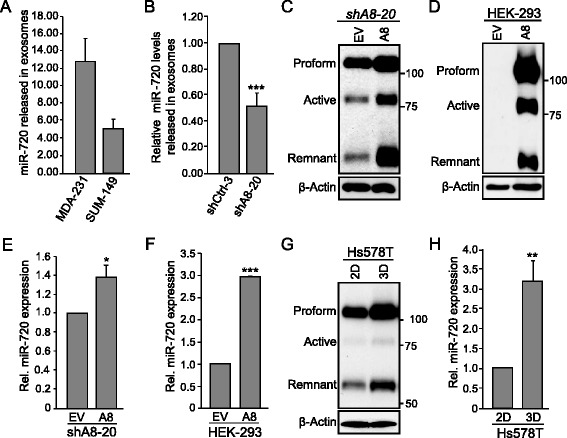
miR-720 is secreted in media and its expression is positively regulated by ADAM8 in TNBC and HEK-293 cells. **a** Cultures of MDA-MB-231 and SUM-149 cells at 50–70 % confluency were grown either in serum-free or exosome-depleted FBS media, respectively. After 72 h, supernatants were collected, exosomes purified, and RNA isolated and subjected to RT-qPCR analysis for miR-720. miRNA expression levels are given as mean ± SEM from two independent experiments. **b** Exosomes were purified from control shRNA (*shCtrl-3*) and a stable clone of MDA-MB-231 cells expressing *ADAM8* shRNA (*shA8-20*) and relative miR-720 expression levels determined, as described for MDA-MB-231 cells in part (**a**). miR-720 levels in exosomes released by the two lines are given as the mean ± SEM from two independent experiments. **c**–**f** MDA-MB-231 shA8-20 and HEK-293 cells were transfected in six-well plates with 4 or 2 μg, respectively, of empty vector (*EV*) or an ADAM8 expression vector (*A8*) for 48 h. WCEs from shA8-20 (**c**) and HEK-293 (**d**) cells were subjected to Western blotting for ADAM8 and β-actin, as above. Representative blots are shown (*n* = 3). RNAs from shA8-20 (**e**) and HEK-293 (**f**) cells were subjected to RT-qPCR for miR-720 levels, as in Fig. 1 f. Control condition (EV) is set to 1 (mean ± SD from three independent experiments). **g**, **h** Hs578T cells were cultured on plastic (two-dimensional (*2D*)) or in suspension (three-dimensional (*3D*)) for 48 h, and WCEs and RNA isolated. WCEs were analyzed by Western blotting for ADAM8 and β-actin (**g**), and RNA subjected to RT-qPCR for miR-720 levels as above (**h**). EV is set to 1 (mean ± SD from three independent experiments). *P < 0.05, **P < 0.01, ***P < 0.001, Student’s *t* test

We next sought to confirm that miR-720 is secreted from breast cancer cells, as reported previously, and that its secretion is regulated by ADAM8. To address this question, we isolated exosomes released into the media by MDA-MB-231 and SUM-149 TNBC cells, extracted miRNA and subjected the RNA to RT-qPCR for miR-720. Consistent with the findings of Pigati et al. [45], these two breast cancer cells lines were shown to release miR-720 (Fig. 2a). To confirm that miR-720 release is controlled by ADAM8, exosomal levels of miR-720 in cultures of MDA-MB-231 cells with shCtrl RNA (shCtrl-3) versus shA8-20 with stable shRNA knockdown of ADAM8 [24] were compared. The release of miR-720 into the exosomes by shA8-20 cells was decreased relative to the shCtrl-3 cells (Fig. 2b). Thus, TNBC cells release miR-720 in an ADAM8-dependent manner.

To further assess the role of ADAM8 in regulation of miR-720, we ectopically expressed full-length ADAM8 in cell lines that either express low levels of ADAM8 or are ADAM8-negative. In particular, MDA-MB-231 cells with stable ADAM8 shRNA knockdown (shA8-20) and HEK-293 that do not express ADAM8 [24] were selected. WCEs and RNA were isolated 48 h after transfection with an ADAM8 expression vector or with empty vector (EV) DNA as control. Western blotting confirmed the ectopic expression and processing of ADAM8 in these cells (Fig. 2c, d). As judged by RT-qPCR, ADAM8 expression led to an increase in miR-720 level of 1.4-fold in shA8-20 cells (Fig. 2e) and of threefold in HEK-293 cells (Fig. 2f). This was consistent with the relative levels of ectopic ADAM8 overexpression and transfection efficiencies of these lines. Previously, we showed that culturing Hs578T TNBC cells in suspension (3D condition) increased the levels and processing of ADAM8 compared to growth on plastic (two-dimensional (2D) condition) [24]. Thus, we asked whether this increase in endogenous ADAM8 expression affects miR-720 levels. As seen previously, significant increases in ADAM8 levels were detected when Hs578T cells were grown in suspension compared to those grown on plastic (Fig. 2g). RT-qPCR analysis of RNA collected from cells after 48 h of culture revealed that miR-720 levels were elevated more than threefold in Hs578T cells in 3D versus 2D cultures (Fig. 2h). Thus, endogenous or ectopic expression of ADAM8 leads to increased miR-720 levels.

### The MP domain of ADAM8 is not essential to induce miR-720 expression

**Fig. 3:**
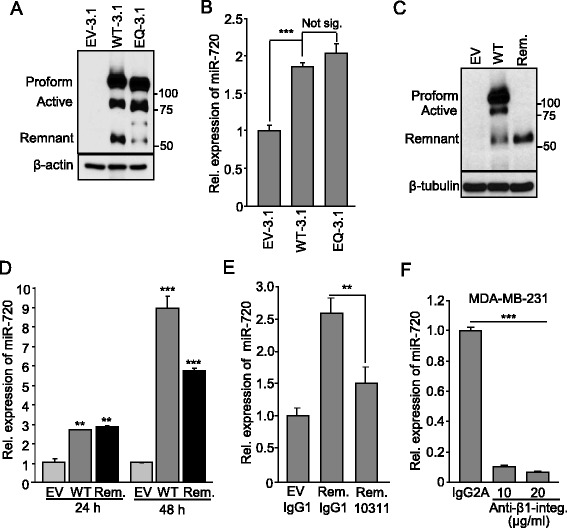
ADAM8 DI domain induces expression of miR-720 via β1-integrin signaling. **a**, **b** HEK-293 cells were transfected in six-well plates with 2 μg of either pcDNA3.1 myc-his vector (*EV-3.1*), or vectors expressing ADAM8 (*WT-3.1* or *EQ-3.1*) for 48 h. WCEs and RNA were then collected. Samples of WCEs were subjected to Western blotting for ADAM8 and β-actin, as in Fig. 1. A representative blot is shown (*n* = 3) (**a**). RNAs were subjected to RT-qPCR for measurement of miR-720 levels and values presented relative to the control condition (EV-3.1), which is set to 1 (mean ± SD from three independent experiments), as above (**b**). **c**, **d** HEK-293 cells were transfected as above with EV DNA or with vectors expressing ADAM8 WT or remnant form (*Rem.*) for 24 h and 48 h. WCEs harvested 48 h after transfection were analyzed for ADAM8 and β-tubulin. A representative blot is shown (*n* = 3) (**c**). RNA was subjected to RT-qPCR for measurement of miR-720 levels. EV control condition is set to 1 (mean ± SD from three independent experiments) (**d**). **e** HEK-293 cells were transfected in 12-well plates with a vector expressing the ADAM8 remnant form (*Rem.*). After 24 h, the transfected cells were treated with 20 μg/ml ADAM8 antibody MAB10311, which targets the CRD/ELD domains and inhibits DI activity [24], or isotype-matched control IgG1 for another 24 h. RNA was extracted and subjected to RT-qPCR analysis for miR-720 levels. Control condition (EV + IgG1) is set to 1 (mean ± SD from three independent experiments). **f** MDA-MB-231 cells were treated with 10 or 20 μg/ml β1-integrin (*Anti-β1-integ.*) antagonist antibody or isotype-matched control IgG2A for 24 h, and miR-720 levels determined by RT-qPCR analysis. Control condition (IgG2A) is set to 1 (mean ± SD from three independent experiments). **P < 0.01, ***P < 0.001, Student’s *t* test. *Not sig.* not significant, *Rel.* relative

To determine how ADAM8 regulates miR-720 expression, we first tested the role of its MP domain using vectors that express either an ADAM8 protein that is catalytically inactive or lacking the MP domain. Previously, it has been reported that a Glu (E330) to Gln (Q330) mutation (EQ mutation) in the catalytic MP domain of mouse ADAM8 led to a proteolytically inactive protein [22]. We demonstrated that human ADAM8 with analogous EQ mutation was similarly inactive, as judged by its inability to shed a 29-kDa CD23 fragment following co-transfection into HEK-293 cells [28]. Here, HEK-293 cells were transfected with vectors expressing either WT ADAM8 (WT-3.1) or EQ ADAM8 (EQ-3.1) or empty pcDNA3.1 myc/his EV-3.1 vector DNA as control. Western blot analysis confirmed the expression of WT and EQ mutant ADAM8 protein (Fig. 3a). As found previously [28], a band was seen with EQ-3.1 that ran somewhat faster than the 90-kDa active ADAM8 seen with the WT-3.1 vector, presumably resulting from non-autocatalytic proteolysis of the EQ mutant proform. An essentially equal increase in miR-720 levels was triggered by the WT-3.1 and EQ-3.1 ADAM8 proteins (Fig. 3b), suggesting that the activity of the MP domain of ADAM8 is not required for miR-720 induction. To confirm that the metalloproteinase activity was not essential, we tested the ability of the ADAM8 remnant form, which lacks the MP domain, to induce miR-720. HEK-293 cells were transfected with pcDNA3 vectors expressing either WT ADAM8 or its remnant form, or control EV pcDNA3 DNA for 24 or 48 h. Western blot analysis confirmed substantial expression of the remnant form (Fig. 3c). At 24 h, the vectors expressing either the WT or remnant forms of ADAM8 induced the expression of miR-720 equally (approximately threefold each) compared to the EV control condition (Fig. 3d). At 48 h, a further increase in miR-720 levels was observed with both forms, but was more pronounced with the WT likely due to expression of both active and remnant ADAM8 (Fig. 3d). Thus, the remnant form of ADAM8, which lacks metalloproteinase activity, effectively induces miR-720 expression, suggesting the MP domain of ADAM8 is not involved in the induction.

### miR-720 induction is mediated by the DI/CRD/ELD region of ADAM8 via activation of a β1-integrin/ERK cascade

The MP and DI domains have been shown to constitute two independent functional cores of ADAM8 activity [18]. The DI domain and the CRD/ELD portions of ADAM proteins have been suggested to form an essential binding domain [48] for interaction with integrins [49, 50]. Since we have shown that ADAM8 is required for β1-integrin activation [24], which is known to participate in adhesion of breast cancer cells to the endothelium [51, 52], we investigated the potential role of the DI/CRD/ELD domains in miR-720 activation. The effects of treatment with the MAB10311 monoclonal antibody that specifically recognizes the CRD/ELD region of ADAM8 on miR-720 expression were tested. HEK-293 cells ectopically expressing the remnant form of ADAM8 or EV DNA were incubated with 20 μg/ml of either ADAM8 antibody MAB10311 or isotype-matched control IgG1 (MAB002). After 24 h of treatment, RNA was extracted and RT-qPCR analysis performed to measure miR-720 levels (Fig. 3e). The induction of miR-720 by the remnant form was significantly inhibited (~40 % inhibition) upon addition of the anti-ADAM8 CRD/ELD antibody. To determine whether direct inhibition of β1-integrin signaling was sufficient to reduce the levels of miR-720 expression, MDA-MB-231 cells were treated for 24 h with 10 or 20 μg/ml of an antagonist anti-human β1-integrin antibody or with an isotype-matched IgG2A. The levels of miR-720 were significantly decreased by approximately 10-fold upon treatment with the β1-integrin antibody when compared to the control (Fig. 3f). Altogether, these findings indicate that the DI/CRD/ELD domains of ADAM8 play a key role in the regulation of miR-720 via activation of the β1-integrin signaling pathway.

**Fig. 4:**
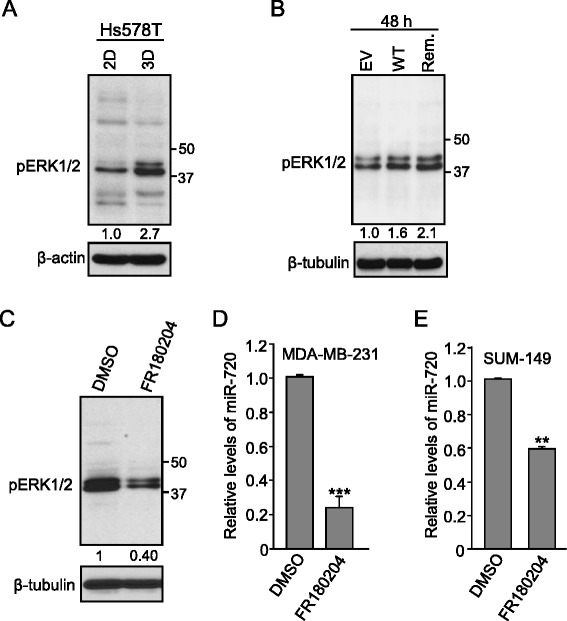
ADAM8 promotes ERK activation, which is required for miR-720 expression. **a** Hs578T cells were cultured on plastic (two-dimensional (*2D*)) or in suspension (three-dimensional (*3D*)) for 48 h. WCEs and RNA were then isolated. WCEs were subjected to Western blotting for the ERK1/2 phosphorylated form (*pERK1/2*) and β-actin. The data from this and two other replicate experiments were quantified. The value of pERK1/2 in the 3D culture is given relative to the 2D culture, which was set to 1.0. **b** HEK-293 cells were transfected as above with empty vector (*EV*) or with vectors expressing ADAM8 (*WT*) or remnant form (*Rem.*) for 48 h. WCEs were analyzed for pERK1/2 and β-tubulin. The data from this and two independent replicate experiments were quantified. The value of pERK1/2 in the control EV DNA was set to 1.0. **c**, **d** MDA-MB-231 cells were cultured in serum-free medium for 24 h, and treated with 25 μM of ERK inhibitor FR180204 or control carrier DMSO for an additional 24 h. WCEs and RNA were collected. WCEs were subjected to Western blotting for pERK1/2 and β-tubulin (**c**). The data from this and two independent replicate experiments were quantified. The value of pERK1/2 in the DMSO sample was set to 1.0. RNA was subjected to RT-qPCR analysis and miR-720 levels were determined. The control condition (DMSO) was set to 1.0 (mean ± SD from three independent experiments) (**d**). **e** SUM-149 cells were cultured in serum-free medium for 24 h, treated with 25 μM of ERK inhibitor FR180204 or carrier DMSO as a control for an additional 24 h. RNA was subjected to RT-qPCR analysis for miR-720 levels. The DMSO control condition was set to 1.0 (mean ± SD from three independent experiments). **P < 0.01, ***P < 0.001, Student’s *t* test

Overexpression of ADAM8 has been shown to also activate ERK/MAPK signaling in osteoclasts [53]. To determine whether ADAM8 leads to ERK activation in breast cancer cells, we tested ERK1/2 phosphorylation (p44/p42) levels in Hs578T cells that overexpress ADAM8 naturally when cultured under 3D conditions versus 2D, as shown above in Fig. 2g. WCEs from Hs578T cells cultured in 2D versus 3D were subjected to Western blotting. In cells grown under 3D conditions, where cells display elevated expression and processing of ADAM8 (Fig. 2g), we observed a 2.7-fold increase in the levels of pERK1/2 (Fig. 4a). A similar increase in levels of pERK1/2 was observed in HEK cells transfected with either WT or the remnant form of ADAM8 (Fig. 4b), indicating that the MP domain is not required for activation of ERK signaling, consistent with the induction of miR-720 observed above. Interestingly, β1-integrin has also been shown to signal predominantly through the recruitment and activation of Src-family kinases, which then activate a downstream cascade including ERK/MAPK and JNK kinases [54]. As phosphorylation of ERK has been implicated in the induction of both cell migration and anchorage-independent growth in breast cancer [55], we asked whether ERK signaling was involved in miR-720 regulation. To address this question, MDA-MB-231 cells were treated with either 25 μM of the ERK specific inhibitor FR180204 [56], or an equivalent volume of DMSO. After 24 h, WCEs and RNA were isolated. As expected, treatment with FR180204 caused a twofold decrease in pERK1/2 levels in comparison to DMSO (Fig. 4c). Notably, a significant decrease in miR-720 expression was observed upon ERK inhibition (Fig. 4d). Similarly, treatment of SUM-149 cells with FR180204 caused a 40 % drop in miR-720 expression (Fig. 4e). Thus, the induction of miR-720 by ADAM8 is mediated via activation of a β1-integrin to ERK1/2 signaling pathway in TNBC cells.

### miR-720 mediates ADAM8-induced aggressive phenotype in TNBC cells

**Fig. 5:**
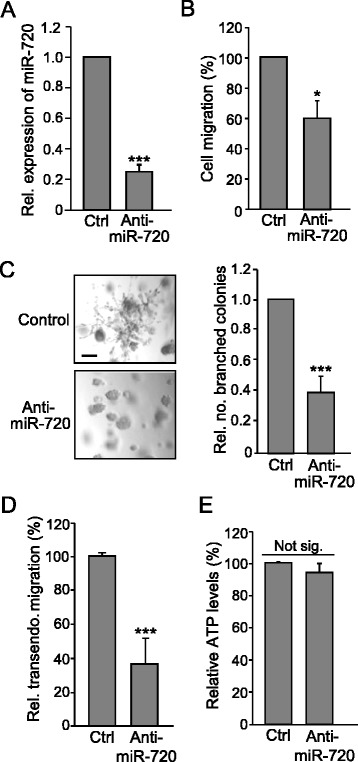
miR-720 maintains the migratory and invasive properties of MDA-MB-231 cells. MDA-MB-231 cells were transfected with 50 nM anti-miR-720 or anti-miR-negative control (*Ctrl*) for 48 h. Either RNA was isolated (**a**) or transfected cells subjected to functional assays (**b**–**e**). **a** miR-720 levels. RNA was subjected to RT-qPCR for miR-720. Control condition (Ctrl) was set to 1.0 (mean ± SD from three independent experiments). **b** Migration assay. Transfected cells were subjected to a migration assay in a Boyden chamber over a 24-h period. The cells that migrated through the chamber were quantified using crystal violet staining. Ctrl was set to 100 % (mean ± SD from three independent experiments). **c** Matrigel assay. Transfected cells were grown in Matrigel for 11–15 days and then photographed using a Nikon eclipse TS100 microscope at 10× magnification. *Scale bar* = 100 μm. Representative images of two independent experiments with similar results are shown (*left panels*). The number of branched colonies were counted. The count in the control sample was set to 1.0 (*right panel*). **d** Transendothelial migration assay. Transfected MDA-MB-231 cells were plated in a Boyden chamber that had been coated with a confluent monolayer of HUVEC cells, and the cancer cells that invaded through the HUVEC layer over a 24-h period were quantified using crystal violet staining. Ctrl value for transendothelial (*transendo.*) migration was set to 100 % (mean ± SD from three independent experiments) and relative (*Rel.*) migration values given. **e** ATP assay. Transfected cells were grown for 24 h and ATP levels were measured using an ATPlite 1Step assay. Ctrl was set to 100 % (mean ± SD from three independent experiments). *P < 0.05, ***P < 0.001, Student’s *t* test. *Not sig.* not significant

**Fig. 6:**
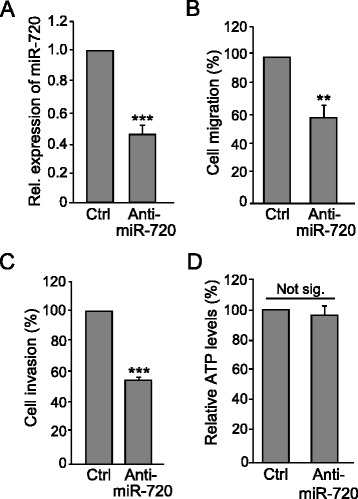
miR-720 maintains the migratory and invasive properties of SUM-149 cells. SUM-149 cells were transfected with 50 nM anti-miR-720 or anti-miR-negative control (*Ctrl*) for 48 h, and either RNA isolated or cells subjected to functional assays. **a** miR-720 levels were determined using RT-qPCR, as in Fig. 5a. **b** Migration assay. Transfected cells that migrated through a Boyden chamber over a 24-h period were quantified as in Fig. 5b. Ctrl was set to 100 % (mean ± SD from three independent experiments). **c** Invasion assay. Transfected cells that invaded through a Matrigel-coated Boyden chamber in a 12-h period were quantified. Ctrl was set to 100 % (mean ± SD from three independent experiments). **d** ATP assay. Transfected cells were grown for 24 h and ATP levels measured using an ATPlite 1Step assay. Ctrl was set to 100 % (mean ± SD from three independent experiments). **P < 0.01, ***P < 0.001, Student’s *t* test. *Not sig.* not significant, *Rel*. relative

As ADAM8 promotes the migratory and invasive phenotype of TNBC cells [24], the role of miR-720 as a mediator of these aggressive properties was tested using a specific anti-miR. MDA-MB-231 cells were transfected with 50 nM anti-miR-720 for 48 h and a 70 % decrease in miR-720 levels was measured (Fig. 5a), leading to a 40 % decrease in MDA-MB-231 cell migration in a Boyden chamber assay (Fig. 5b). Furthermore, miR-720 knockdown robustly inhibited the capacity of MDA-MB-231 cells to form characteristic branching colonies in Matrigel (Fig. 5c, left panel). The numbers of branched colonies were counted and a ~60 % reduction was seen in three independent experiments (Fig. 5c, right panel). To determine whether miR-720 was likely playing a role in β1-integrin-mediated transmigration of cancer cells through the endothelial layer of blood vessel walls [57], the effects of miR-720 knockdown on transendothelial migration were monitored. MDA-MB-231 cells that had been treated with 50 nM anti-miR-720 for 48 h were plated over a HUVEC monolayer in a Boyden chamber and the number of cells that migrated through the endothelial barrier measured after 24 h. The capacity of MDA-MB-231 cells to transmigrate through the endothelial cell layer was reduced by 60 % after miR-720 knockdown (Fig. 5d). In contrast, knockdown of miR-720 over a 24-h period did not decrease 2D growth of MDA-MB-231 cells (Fig. 5e), consistent with our previous findings that growth of TNBC cells on plastic was unaffected by knockdown of ADAM8 [24]. To extend these observations to a second TNBC line, the roles of miR-720 in SUM-149 cells were evaluated. Knockdown of miR-720 (Fig. 6a) resulted in a 40 % decrease in the ability of SUM-149 cells to migrate (Fig. 6b) and to invade through Matrigel (Fig. 6c), while 2D growth was not affected (Fig. 6d).

**Fig. 7:**
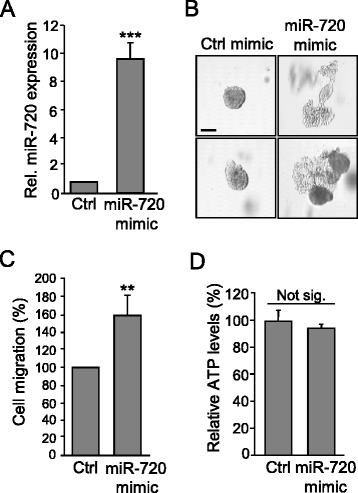
Ectopic expression of miR-720 restores the migratory and invasive abilities of MDA-MB-231 cells with stable knockdown of ADAM8. MDA-MB-231 shA8-20 clonal cells, with stable ADAM8 knockdown, were transfected in six-well plates with 200 pmol of control mimic (*Ctrl*) or miR-720 mimic. After 48 h, either RNAs were isolated or cells subjected to functional assays. **a** miR-720 levels were determined by RT-qPCR. The control mimic (Ctrl) was set to 1 (mean ± SD from three independent experiments). **b** Transfected MDA-MB-231 shA8-20 cells were plated in Matrigel and photographed at 10× magnification after 11–15 days of culture. Representative images of two independent experiments with similar results are shown. *Scale bar* = 100 μm. **c** Transfected cells were subjected to a Boyden chamber migration assay for 24 h, as in Fig. 5. Ctrl was set to 100 % (mean ± SD from three independent experiments). **d** Transfected cells were subjected to an ATP assay following growth on plastic for 24 h. Ctrl was set to 100 % (mean ± SD from three independent experiments). **P < 0.01, ***P < 0.001, Student’s *t* test. *Not sig.* not significant, *Rel*. relative

Finally, to test whether addition of miR-720 was sufficient to overcome the loss of the aggressive phenotype of TNBC cells upon ADAM8 depletion, cultures of shA8-20 MDA-MB-231 cells stably expressing an *ADAM8* shRNA were transfected with 200 pmol of a miR-720 mimic or control miRNA. Expression of the miR-720 mimic resulted in a 10-fold increase in miR-720 levels compared to the control (Fig. 7a). Notably, miR-720 expression was sufficient to profoundly increase the formation of branched colonies from shA8-20 MDA-MB-231 cells in a Matrigel 3D-outgrowth assay (Fig. 7b), and led to more than a 50 % increase in their ability to migrate (Fig. 7c). As expected, growth of the shA8-20 cells on plastic was unaffected by expression of the miR-720 mimic (Fig. 7d). Thus, the ADAM8 downstream target miR-720 participates in many aspects of the invasive and migratory properties of TNBC cells, suggesting that miR-720 is an important mediator of the aggressive properties of TNBC cells induced by ADAM8.

### miR-720 is detected in the plasma of mice bearing ADAM8-positive TNBC tumors

**Fig. 8:**
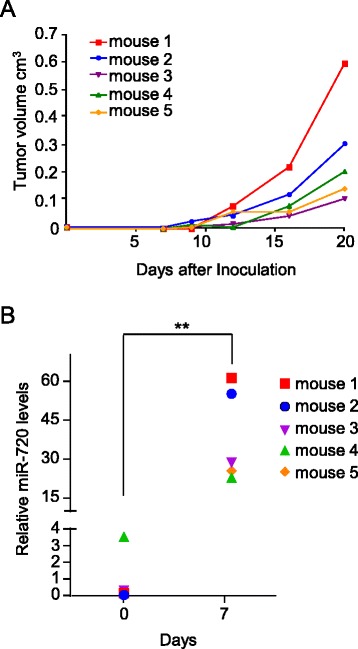
miR-720 is detected in the plasma of mice before tumors become palpable. MDA-MB-231 shCtrl-3 cells (2.5 × 10^6^ in 40 μl 50 % Matrigel) were implanted on day 1 into the mammary fat pad of female mice (*n* = 5). Tumor volume was measured on the indicated days. Blood was collected on the day prior to implantation and on day 7 by submandibular bleeding and centrifuged to obtain clear plasma. Total RNA was collected from plasma samples and miR-720 levels were determined using RT-qPCR. **a** Tumor growth in individual mice from day 1 to day 21. Tumor volume (cm^3^) is presented. **b** Relative miR-720 levels in plasma of the individual mice at day 0 and day 7. **P < 0.005, Student’s *t* test

To begin to evaluate the potential of using miR-720 as a biomarker for ADAM8-expressing TNBCs, an orthotopic mouse model was performed using control MDA-MB-231 cells (shCtrl-3 clone), which express high levels of ADAM8 and miR-720. In our preliminary analysis, the plasma levels of miR-720 appeared to vary between individual mice (data not shown). Thus, plasma was collected from each 6-week-old female NOD/SCID mouse (*n* = 5) a day before TNBC cell inoculation into the mammary fat pad, and used to establish baseline miR-720 levels for each individual mouse. On day 1, 2.5 × 10^6^ shCtrl-3 MDA-MB-231 cells were implanted into the fourth inguinal mammary fat pad and primary tumor growth was monitored by palpation and caliper measurement twice a week thereafter (Fig. 8a). Little growth of the tumors was detected within the first 7–9 days, as reported previously [24]. Plasma was collected 7 days after cell implantation when the tumors were barely palpable. Tumor growth was followed for a total of 21 days (Fig. 8a). A significant increase in miR-720 levels of about 40-fold was detected in the blood of mice at the 7-day time point (Fig. 8b) and remained elevated above 10-fold to day 21 (data not shown). It was interesting to note that the mice with the two fastest growing tumors (mouse 1 and mouse 2) had the highest miR-720 levels at day 7. Thus, substantial increases in miR-720 levels can be detected early, prior to the increase in tumor growth when tumor sizes are fairly small.

### miR-720 levels are elevated in serum of TNBC patients with high ADAM8 levels

**Fig. 9:**
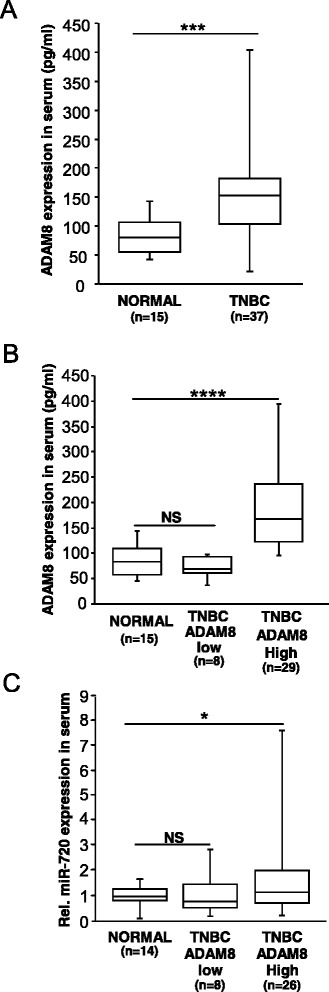
High levels of miR-720 are detected in the serum of triple-negative breast cancer (*TNBC*) patients with higher ADAM8 levels. Sera were obtained from 37 TNBC patients and 15 healthy individuals and analyzed for protein levels of ADAM8 and RNA levels of miR-720. **a**, **b** ADAM8 protein levels were measured by ELISA in serum samples from TNBC patients and healthy individuals (**a**). TNBC patients were separated based on their ADAM8 levels: high ADAM8 > 100 pg/ml and low ADAM8 < 100 pg/ml (**b**). **c** Total RNA was collected from serum samples and miR-720 levels were determined using RT-qPCR. Relative miR-720 levels in serum of TNBC patients with high ADAM8, low ADAM8 and normal individuals are presented. *P <0.05, ***P < 0.001, ****P < 0.0001, Welch’s *t* test. *NS* not significant

The MP domain alone or the entire ectodomain of ADAM8 has been shown to be secreted from cells expressing ADAM8 [22]. This soluble fraction of ADAM8 was recently found to be shed from malignant breast tumors into the blood of patients [24]. Given that miR-720 levels were significantly higher in the blood of mice bearing ADAM8-positive TNBC tumors, we next asked whether miR-720 levels are elevated in the serum of TNBC patients with detectable ADAM8 in the blood versus normal individuals. Serum samples were obtained from consenting TNBC patients taken at the time of diagnosis, before any treatment was provided, and from normal healthy individuals. Samples were first analyzed for levels of soluble ADAM8 protein shed into the blood using an ELISA kit (R&D Systems). ADAM8 levels were significantly higher in serum samples of TNBC patients in comparison to normal healthy individuals (Fig. 9a). Interestingly, 78 % (29/37) of the TNBC patients had ADAM8 levels above the median value observed in healthy individuals, whereas levels of eight TNBC patient serum samples were below the medium value (Fig. 9b). Next, RNA was extracted from the serum samples using a Macherey-Nagel Nucleospin miRNA plasma kit and subjected to RT-PCR for miR-720. Unfortunately, RNA yield and quality from three of the samples were poor as judged by RNA integrity analysis using an Agilent bioanalyzer, and these samples had to be excluded from the analysis. hsa-miR-16 was used to normalize the data as its levels remained unchanged in normal versus patient samples (Additional file 3: Figure S2), similar to previous reports by others in healthy individuals versus breast cancer patients [58]. As seen in Fig. 9c, levels of miR-720 were significantly higher in patient samples with elevated amounts of ADAM8, whereas normal healthy individuals and TNBC patients with comparably low ADAM8 levels displayed low levels of miR-720. This increase was not correlated with tumor size (Additional file 3: Figure S2). Thus, higher levels of miR-720 are found in the serum of TNBC patients with high ADAM8 serum levels.

## Discussion

We show for the first time that ADAM8 signaling regulates a miRNA subset and that miR-720 specifically plays a critical role in the ability of this transmembrane protein to promote an aggressive phenotype in TNBC cells. In MDA-MB-231 and SUM-149 TNBC cells in culture, knockdown of ADAM8 led to decreased levels of miR-720 while ADAM8 ectopic overexpression increased miR-720 expression. Surprisingly, the induction of miR-720 did not require the metalloproteinase activity of ADAM8, as ectopic expression of either an ADAM8 mutant with a catalytically inactive MP domain or the remnant form lacking the MP domain was essentially as effective as full-length ADAM8 in promoting miR-720 expression. An antibody against the CRD/ELD domain that inhibits the DI activity of ADAM8 [24] or an antagonist antibody against β1-integrin significantly reduced miR-720 levels, mapping the activity to the DI/CRD/ELD domains of ADAM8. The addition of an ERK-specific inhibitor significantly decreased the levels of miR-720. Together, these findings implicate activation of β1-integrin signaling by the DI/CRD/ELD region of ADAM8, and downstream activation of the ERK pathway in miR-720 induction. Significantly, knockdown of miR-720 using an antagomiR decreased the migratory and invasive phenotype of TNBC cells, whereas ectopic miR-720 expression restored these properties. We confirmed that miR-720 is secreted from TNBC cells, consistent with observations made in other breast cancer lines [45], and increased miR-720 levels were detected in the blood of mice 7 days following orthotopic implantation of ADAM8-positive TNBC cells, when tumors were barely palpable. Higher levels of miR-720 were detected in the blood of TNBC patients with elevated amounts of circulating ADAM8. Thus, miR-720 is an essential mediator of ADAM8 in the promotion of the aggressive phenotype of TNBC cells, and as a secreted factor has the potential to function as a biomarker for early detection of ADAM8-positive recurrent TNBCs.

While miRNAs have been shown to play a vital role in breast cancer development and to regulate the functions of a number of critical genes [33, 59, 60], very little is known about the stimuli and processes regulating the biogenesis of miRNAs themselves. Butz et al. [61] and Wang et al. [62] elucidated complex pathways downstream of TGF-β1 and c-MYC signaling, respectively, that modulated miRNA processing. For miR-720, work by Ragusa et al. [63] suggested that its expression may be induced downstream of a MAPK/ERK pathway in colorectal cancer cells, although the mechanism of activation was not elucidated. Our results agree with and extend these findings, showing for the first time that ADAM8 initiates the induction of miR-720 via activation of the β1-integrin/ERK signaling cascade to promote migration and invasion in breast cancer cells. These findings shed light into the complex mechanism by which ADAM8 promotes aggressive phenotype of TNBCs. Many studies have implicated miR-720 in transformed phenotype or suggested its use in cancer diagnosis or as a prognostic marker for cancer [46, 64, 65]. As discussed above, Lerebours et al. [25] identified miR-720 in a set of five miRNAs that can serve as predictive markers of poor prognosis in patients with IBC, which we have found also frequently express ADAM8 (data not shown). Similarly, Park and coworkers reported upregulation of miR-720 in blood of patients with metastatic ER+/HER2- breast cancer [66]. Consistently, miR-720 is upregulated in a variety of other tumors including colorectal and bladder cancers, malignant melanoma, renal cell carcinoma and multiple myeloma [46, 63, 65, 67–71]. In contrast, Li et al. [72] reported that miR-720 prevented a more aggressive phenotype of breast cancer cells, specifically via repression of synthesis of the EMT marker TWIST1 in MDA-MB-231 breast cancer cells. While potentially interesting, the expression levels of TWIST1, miR-720, and N-cadherin in the stock of MDA-MB-231 cells used in this study are inconsistent with NIH criteria established for this line, as judged by analysis of the NCI-60 cell line panel [73, 74].

In addition to miR-720, many of the 68 miRNAs modulated by ADAM8 have been found to be upregulated in breast cancer or previously implicated in tumorigenesis (such as miR-19a, miR-106b, miR-181a-2, miR-30a, miR-93, miR-30d, and miR-10b) [37, 40, 75–78]. Out of these 68 miRNAs, two (miR-324 and miR-7) were found to be downregulated by ADAM8. Interestingly, miR-7 is a potent tumor suppressor in breast cancer cells [79] consistent with the observed ability of ADAM8 to repress its expression. Altogether, these data suggest that ADAM8, through β1-integrin and ERK activation, may regulate a large network of oncomiRs, which likely plays additional roles in promoting invasion and metastasis of TNBC tumors expressing high ADAM8 levels.

Stability of miRNAs in both primary tumors and in the blood (plasma and serum samples) makes them attractive potential biomarkers for non-invasive monitoring of cancer recurrence and for evaluating treatment efficacy. Recent studies have shown the importance of miRNAs as biomarkers in breast cancer [80–87]. In particular, the level of miR-210 was found to be a good indicator of the sensitivity of breast cancer patients to trastuzumab [80]. We have shown that levels of miR-720 are increased in the blood of mice bearing barely palpable ADAM8-positive tumors. Furthermore, elevated levels of miR-720 were seen in the serum of TNBC patients with high amounts of soluble ADAM8 protein. Although the miR-720 levels in serum of TNBC patients versus healthy individuals were statistically significant, they were not as elevated as the ADAM8 protein. This finding suggests that miR-720 needs to be used along with other tumor markers for detection of disease, and that miR-720 might be a better marker for recurrence where its levels in the serum under a disease-free state for a patient can be established. Notably, other miRNAs found modulated by ADAM8 in this study have been recently reported to help predict breast cancer risk and tumor relapse in TNBC patients [88, 89]. For example, miR-18b, miR-20a, and miR-30d have been reported to be highly expressed in the serum of relapsing TNBC patients [88]. Also miR-195 was upregulated in the serum of breast cancer patients in the study by Mishra et al. [82], and served within a signature for early detection. Lastly, a recent endpoint study of patient serum samples showed that increased miR-720 levels can be detected in the whole blood of breast cancer patients with metastatic disease [66], consistent with our data showing high ADAM8 expression in almost half of all metastases in breast cancer patients [24]. Taken together with our data, these findings suggest that miR-720 holds potential for use as a biomarker along with a group of miRNAs regulated by ADAM8 and associated with metastasis for early diagnosis of recurrent TNBC or as a pharmacodynamic marker for treatment efficacy.

## Conclusions

In summary, we have shown that ADAM8 regulates miR-720 to promote invasion and metastasis of TNBC. ADAM8 induces miR-720 by activation of the β1-integrin/ERK signaling cascade using its DI/CRD/ELD domain, whereas its MP domain is not essential for this induction. Ectopic miR-720 was sufficient to promote an invasive and migratory phenotype in TNBC cells lacking ADAM8, indicating again that miR-720 is a crucial downstream player of the ADAM8 pathway. Lastly, we have shown that elevated levels of miR-720 can be detected in the blood of mice with barely palpable tumors, as well as in the serum of TNBC patients with high amounts of soluble ADAM8, suggesting that miR-720 has potential as a biomarker for TNBCs.

## Additional files

Additional file 1: Table S1. Clinicopathological characteristics of patient samples. Clinicopathological characteristics of 37 breast cancer patients whose serum samples were analyzed for ADAM8 by ELISA and miR-720 levels by RT-qPCR in Fig. 9. *IC* invasive carcinoma. (PPTX 51 kb) Additional file 2: Figure S1. miRNA expression in MDA-MB-231 cells. RNA was isolated from MDA-MB-231 cells and subjected to RT-qPCR analysis to determine the level of expression of the indicated miRNAs. Relative levels of miRNA expression are depicted as mean ± SEM from two independent experiments. (PPTX 90 kb) Additional file 3: Figure S2. Total RNA was isolated from serum samples of TNBC patients or normal individuals and analyzed for miR-720 levels. **A** Figure depicting Pearson’s correlation coefficient plot for miR-720 expression in patient serum versus the size of the tumor in the patient. **B** Relative miR-16 levels in serum of TNBC patients with high ADAM8, low ADAM8 and normal individuals are presented. Welch’s *t* test. *NS* not significant. (PPTX 43 kb)

## Abbreviations

2D: two-dimensional
3D: three-dimensional
ADAM8: a disintegrin and metalloproteinase 8
ATCC: American Type Culture Collection
BL2: basal-like 2
CRD: cysteine-rich domain
CTC: circulating tumor cell
DI: disintegrin
EGF: epidermal growth factor
ELD: epidermal growth factor-like domain
ELISA: enzyme-linked immunosorbent assay
EQ: Glu (E330) to Gln (Q330) mutation in the MP domain of ADAM8
EQ-3.1: pcDNA3.1 myc-his vector carrying EQ ADAM8
ERK: extracellular signal-regulated kinase
ERα: estrogen receptor alpha
EV: empty pcDNA3 vector
EV-3.1: pcDNA3.1 myc-his vector
HER2: human epidermal growth factor receptor 2
HUVEC: human umbilical vein endothelial cell
IBC: inflammatory breast cancer
JNK: c-Jun N-terminal kinase
MAPK: mitogen-activated protein kinase
miRNA: microRNA
MP: metalloproteinase
NOD/SCID: nonobese diabetic/severe combined immunodeficient
OncomiR: oncogenic microRNA
PCR: polymerase chain reaction
pERK1/2: ERK1/2 phosphorylated form
PRO: prodomain
RIN: RNA integrity number
RT: reverse transcriptase
RT-qPCR: quantitative reverse transcription polymerase chain reaction
shA8-20: a stable clone of MDA-MB-231 cells expressing *ADAM8* shRNA
shCtrl-3: control short hairpin RNA
shRNA: short hairpin RNA
*siCtrl*: non-silencing control siRNA
siRNAs: short interfering RNAs
TNBC: triple-negative breast cancer
WCE: whole cell extract
WT-3.1: pcDNA3.1 myc-his vector carrying WT ADAM8
