# Supplementary material for: miR-720 is a downstream target of an ADAM8-induced ERK signaling cascade that promotes the migratory and invasive phenotype of triple-negative breast cancer cells
Source: Breast Cancer Res. 2016 Apr 2;18:40. doi: 10.1186/s13058-016-0699-z (PMC4818899; doi:10.1186/s13058-016-0699-z)
Supplement: Additional file 1: Table S1. — Clinicopathological characteristics of patient samples. Clinicopathological characteristics of 37 breast cancer patients whose serum samples were analyzed for ADAM8 by ELISA and miR-720 levels by RT-qPCR in Fig. 9. IC invasive carcinoma. (PPTX 51 kb) [file 13058_2016_699_MOESM1_ESM.pptx]

## Slide 1
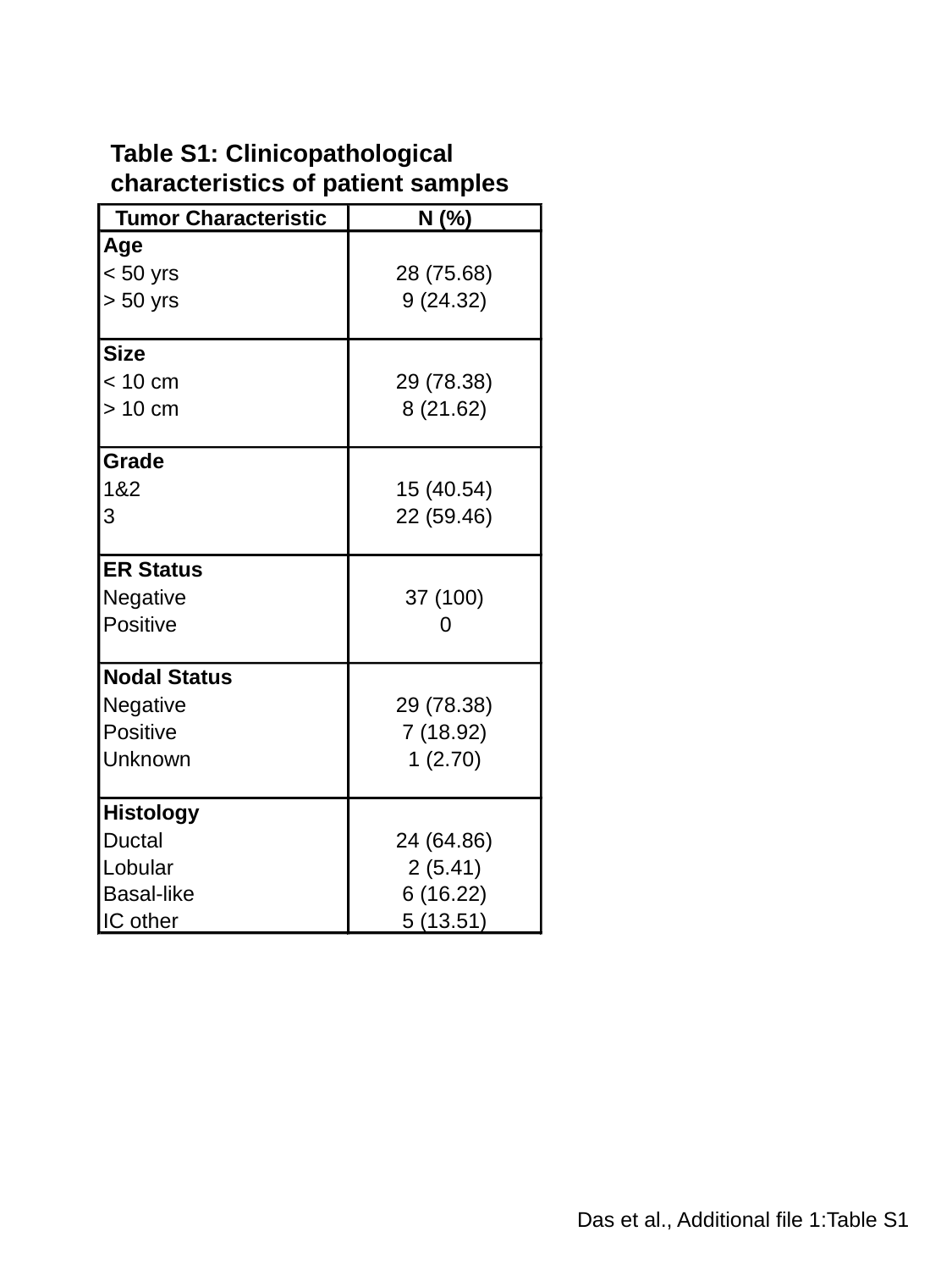

Table S1: Clinicopathological characteristics of patient samples
Tumor Characteristic
N (%)
Age
< 50 yrs
28 (75.68)
> 50 yrs
9 (24.32)
Size
< 10 cm
29 (78.38)
> 10 cm
8 (21.62)
Grade
1&2
15 (40.54)
3
22 (59.46)
ER Status
Negative
37 (100)
Positive
0
Nodal Status
Negative
29 (78.38)
Positive
7 (18.92)
Unknown
1 (2.70)
Histology
Ductal
24 (64.86)
Lobular
2 (5.41)
Basal-like
6 (16.22)
IC other
5 (13.51)
Das et al., Additional file 1:Table S1
