# Supplementary material for: miR-720 is a downstream target of an ADAM8-induced ERK signaling cascade that promotes the migratory and invasive phenotype of triple-negative breast cancer cells
Source: Breast Cancer Res. 2016 Apr 2;18:40. doi: 10.1186/s13058-016-0699-z (PMC4818899; doi:10.1186/s13058-016-0699-z)
Supplement: Additional file 2: Figure S1. — miRNA expression in MDA-MB-231 cells. RNA was isolated from MDA-MB-231 cells and subjected to RT-qPCR analysis to determine the level of expression of the indicated miRNAs. Relative levels of miRNA expression are depicted as mean ± SEM from two independent experiments. (PPTX 90 kb) [file 13058_2016_699_MOESM2_ESM.pptx]

## Slide 1
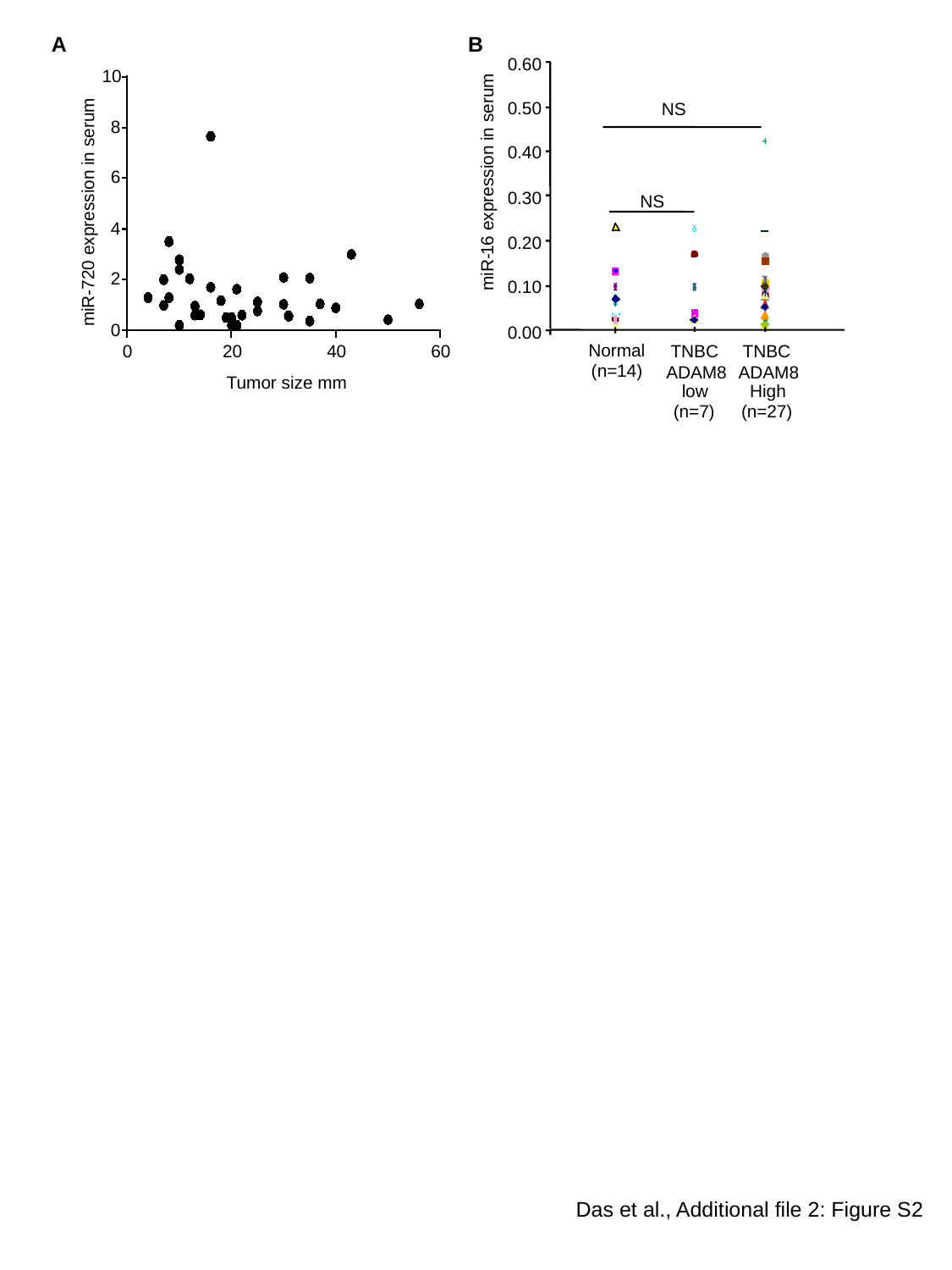

A
10
8
6
miR-720 expression in serum
4
2
0
0
20
40
60
Tumor size mm
B
0.60
0.50
NS
0.40
16 expression in serum
0.30
NS
0.20
-
miR
0.10
0.00
Normal
TNBC
TNBC
(n=14)
ADAM8
ADAM8
low
High
(n=7)
(n=27)
Das et al., Additional file 2: Figure S2
