# Supplementary material for: miR-720 is a downstream target of an ADAM8-induced ERK signaling cascade that promotes the migratory and invasive phenotype of triple-negative breast cancer cells
Source: Breast Cancer Res. 2016 Apr 2;18:40. doi: 10.1186/s13058-016-0699-z (PMC4818899; doi:10.1186/s13058-016-0699-z)
Supplement: Additional file 3: Figure S2. — Total RNA was isolated from serum samples of TNBC patients or normal individuals and analyzed for miR-720 levels. A Figure depicting Pearson’s correlation coefficient plot for miR-720 expression in patient serum versus the size of the tumor in the patient. B Relative miR-16 levels in serum of TNBC patients with high ADAM8, low ADAM8 and normal individuals are presented. Welch’s t test. NS not significant. (PPTX 43 kb) [file 13058_2016_699_MOESM3_ESM.pptx]

## Slide 1
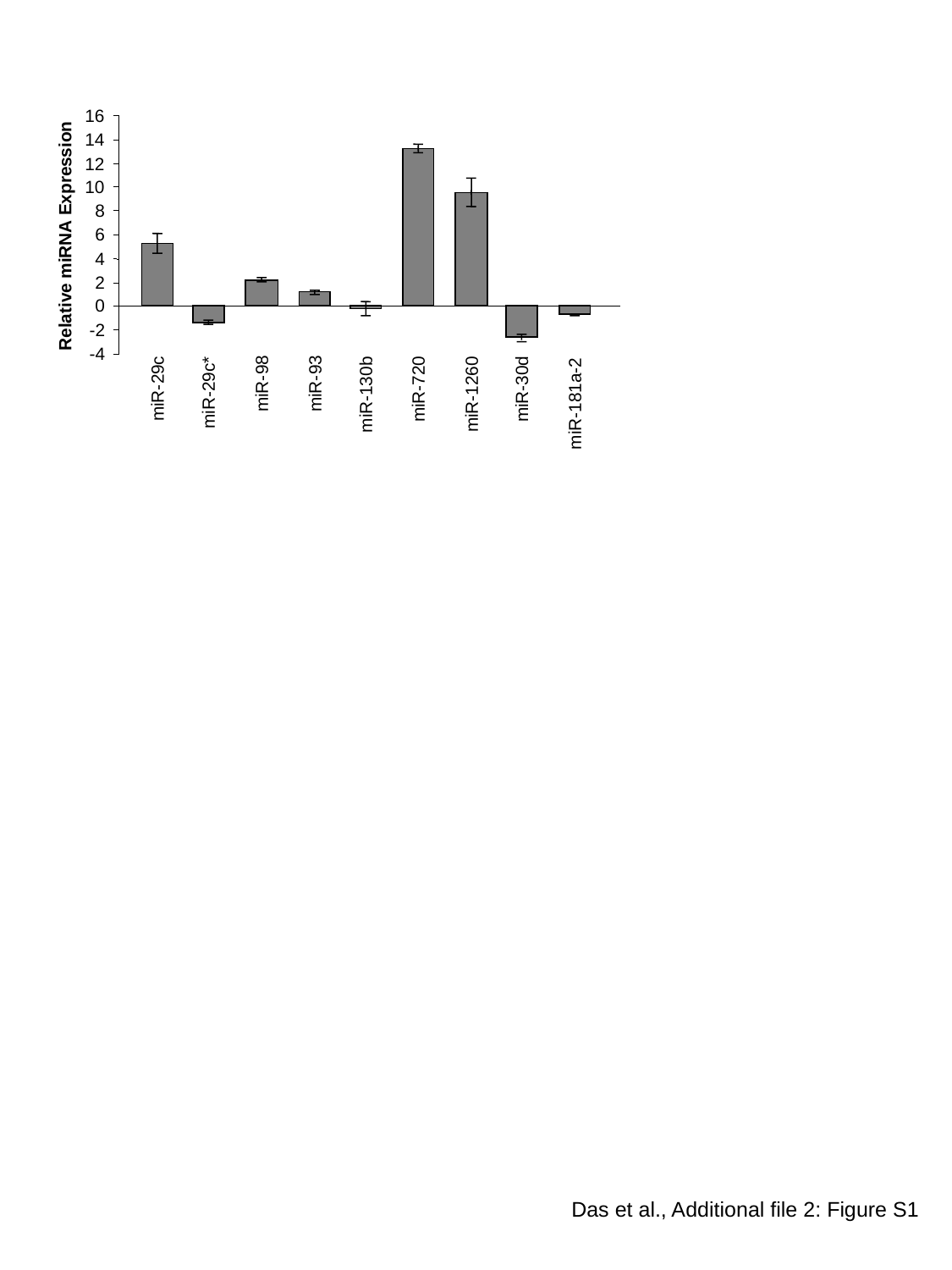

16
14
12
10
8
Relative miRNA Expression
6
4
2
0
-2
-4
miR-98
miR-93
miR-29c
miR-720
miR-30d
miR-29c*
miR-1260
miR-130b
miR-181a-2
Das et al., Additional file 2: Figure S1
